# Rhizosphere mediated growth enhancement using phosphate solubilizing rhizobacteria and their tri-calcium phosphate solubilization activity under pot culture assays in Rice (*Oryza sativa.*)

**DOI:** 10.1016/j.sjbs.2021.05.052

**Published:** 2021-05-24

**Authors:** Renu Gupta, Anshu, Ahmed Noureldeen, Hadeer Darwish

**Affiliations:** a Division of Soil Science and Agriculture Chemistry, Sher-e-Kashmir University of Agricultural Sciences and Technology, Chatha, Jammu, India; b Department of Biology, College of Sciences, Taif University, P.O. Box 11099, Taif 21944, Saudi Arabia; c Department of Biotechnology, College of Sciences, Taif University, P.O. Box 11099, Taif 21944, Saudi Arabia

**Keywords:** Rice, Tricalcium phosphate (TCP), Rhizosphere, Phosphate Solublizing Rhizobacteria (PSRB)

## Abstract

Phosphate solubilizing rhizobacteria are considered as an important alternative to increase the availability of accumulated phosphates through solubilization. These increase the growth of plant by enhancing the efficiency of fixing biological nitrogen. This was studied through a pot experiment involving two Phosphate Solubilizing Rhizobacteria (PSRB) isolates, *Pseudomonas aeruginosa* and *Bacillus subtilis* along with Tri-calcium phosphate (TCP) on availibity of nutrients, biological composition of soil and yield attributes of rice crop at its growth stages. Experiment was laid in factorial completely randomized design (CRD) comprising of eight treatments replicated thrice with two factors viz. factor 1 with or without TCP (1 g^−1^soil) and factor 2 with single or combined inoculation of PSRB isolates. Considerable enhancement in available content of potassium (K), phosphorous (P), nitrogen (N) in soil was found with TCP 1 g^−1^soil (P_1_) and consortium of *Pseudomonas aeruginosa* and *Bacillus subtilis* broth culture at crop growth stages. Highest increase in available N (17.13% and 19.1%), available P (232% and 265%), available K (19.6% and 29.2%) over control were recorded in B_3_ (consortium of *Pseudomonas aeruginosa* and *Bacillus subtilis* broth culture). Similarly, maximum nutrient uptake N (6.4%), P (15.8%) and K (8.9%) were recorded with same treatment. A considerable growth in soil microbial biomass carbon and dehydrogenase activity at crop growth stages was recorded on application of TCP 1 g^−1^soil (P_1_) and consortium of PSRB isolates' *Pseudomonas aeruginosa* and *Bacillus subtilis* (B_3_). Highest increase in microbial biomass carbon (16.4% and 16.5%) and dehydrogenase activity 34.7% and 43.8% over control were recorded in B_3_ (consortium of PSRB isolates *Pseudomonas aeruginosa* and *Bacillus subtilis*) and was found best among all treatments in terms of yield (63.2%) and yield attributes; number of panicles^−1^plant (54.8%), number of grains^−1^panicle (156%) and average panicle length (63.9%).

## 1 Introduction

Phosphorous is one among the major vital macronutrients which is required for proper growth and development of plant species (Sharma et al., 2013). All the necessary biochemical reactions that occur within a plant are dependent upon the availability of phosphorous. It has been observed that majority of agricultural soils contain organic and inorganic forms of P extensively, however, quantity of accessible phosphorous to the plants is meager. Just 0.1 per cent of the overall soil P occurs in a soluble state for plant absorption due to soil fixation and poor solubility of phosphorous in the soil (Pereira and Castro, 2014). It has also been observed that precipitation and immobilization of P inside the soil is usually related to pH of soils. Phosphorous immobilization in alkaline soils is brought about by calcium (Ca) whereas in acidic soils, fixation of P is brought about by aluminum (Al) and Iron (Fe) oxides (Mahdi et al., 2011). Application of P fertilizers is often practiced to maintain crop production. These when applied to soil get converted into lower solubility phosphorous containing compounds for use in small amounts by the plants (Glick, 2012). The efficiency of P fertilizers is increased through their repeated application to soil which in turn affects the diversity of microbes resulting in decreased soil fertility and loss in productivity (Gyaneshwar et al., 2002).

Modern agriculture research promotes sustainable nutrient management with the help of different plant growth promoting rhizobacteria (PGPR) (Bhat et al., 2020, Khanna et al., 2019a, Khanna et al., 2019b, Khanna et al., 2019c, Khanna et al., 2019d, Khanna et al., 2019e, Parray et al., 2016, Sharma et al., 2020). Although different chemical and inorganic nutrients are used to release the stress of plants (Ahmad et al., 2016, Ahmad et al., 2018a, Ahmad et al., 2018b, Ahmad et al., 2018c, Handa et al., 2019, Kaya et al., 2020, Kohli et al., 2018, Siddiqui et al., 2020), an environment friendly microbial mediated P management as a substitute to harmful effects of inorganic chemicals is gaining momentum (Batool and Iqbal, 2019). Rhizosphere inhabiting microbes play a direct vital role to promote the growth and development of plants (Panhwar et al., 2012, Yadav et al., 2016). Among these heterogeneous microbes, phosphate solubilizing microorganisms are very crucial as they promote biological nitrogen fixation, production of phytohormones and various activities related to bio-control (Dugar et al., 2013). Therefore, they arise as an alternate way in the sustainable agriculture. A number of strains of Phosphate Solubilizing Microbes (PSM) has been isolated from the soil. Some bacterial genera which include *Bacillus*, *Erwinia*, *Psedomonas*, *Burkholderia* and *Rhizobium* are considered as the powerful solubilizers of the phosphate (Mamta et al., 2010, Yu et al., 2011, Karpagam and Nagalakshmi, 2014). Phosphate Solubilizing Bacteria fasten the growth and development of plants through P uptake (Liu et al., 2014, Shahid et al., 2012). Survival of Phosphate solubilizing microorganism depends upon their tendency to dwell and multiply in their natural habitats. It is however hard to anticipate the action and effectiveness of the inoculated PSM at a specific site (Gyaneshwar et al., 2002).

The importance of rice as global staple food is highly significant but considerable attention towards the significance of the availability of phosphorous to rice plants is lagging behind with respect to nitrogen nutrition (Islam et al., 2008). The findings of both pot and field-based studies revealed an improvement in the growth and yield of the rice plants that show P nutrient uptake (Son et al., 2007, Panhwar et al., 2011, Vahed et al., 2012). The present study aims at evaluating the performance of phosphate solubilizing rhizobacteria isolates from rice individually and in combination with tricalcium phosphate under pot culture assays.

## 2 Materials and methods

### 2.1 Pot experiment

The pot experiment was performed at factorial CRD design at Division of Soil Science and Agricultural Chemistry SKUAST, Jammu. Pots of dimensions 30*26*17 cm^3^ were filled with polythene containing 6 kg of soil sterilized with 0.5% formaldehyde. Prior to pot filling, 1 g of TCP was thoroughly mixed in 4 Kg sterilized soil to ensure uniform distribution. Roots of seven days old, six rice seedlings were dipped in 10 ml of bacterial broth culture as well as consortium of the two strains (*Pseudomonas aeruginosa* and *Bacillus subtilis*) and kept for 4 h. The remaining suspension was inoculated in digged holes near root zone of rhizosphere along with seedlings in each pot. Two different kinds of soil conditions were prepared, one by mixing 1g of TCP in sterilized soil and second without TCP, for maintaining plantlets, with eight different treatments i: soil (control 1), (ii) soil + TCP1g^−1^soil (control2), (iii) soil + PSRB 1 (*Pseudomonas aeruginosa)*, (iv) Soil + PSRB 2 (*Bacillus subtilis*) (v) Soil + Consortium (50:50) (vi) Soil + TCP + PSRB1 (vii) Soil + TCP + PSRB2 (viii) Soil + TCP + Consortium(50:50). The treatments were replicated thrice. Harvesting was done after four months and different growth parameters of every individual was measured and recorded along with soil samples collected at different growth stages were analyzed for available nutrients and biological activity in soil.

### 2.2 Determination of nitrogen (N), phosphorus (P), potassium (K) and biological activity in soil

Available soil nitrogen was determined by following the method of Subbiah & Asija, 1956. Available phosphorus was estimated by using Olsenn’s extractant method (Olsen *et al*., 1954). 1 N ammonium acetate was used as extractant and the availability of potassium was detected by feeding the extract to flame photometer (Jackson, 1973).

Microbial biomass carbon (MBC) was determined through chloroform fumigation extraction method (Vance et al., 1987). A paired set of 20 g fresh soil samples maintained at 4 °C were taken, Fumigation of one part was performed inside desiccators with chloroform free of ethanol and another was kept under identical conditions without fumigation. These samples were then treated with 0.5 M K_2_SO_4_. The Carbon(C) was measured both in the fumigated and non-fumigated extracts and the difference obtained was used to calculate MBC. Visualizing the rate of formation of triphenyl formazan from triphenyl tetrazolium was used to measure the dehydrogenase activity in soil (Casida *et al*. 1964). Colorimetric estimation of *p*-nitrophenol was used to determine phosphatase activity in soil (Tabatabai and Bremner, 1969).

### 2.3 Determination of yield attributes and plant nutrient uptake in rice

Plant samples were collected from each pot at harvest stage in first week of November and yield attributes were recorded as number of panicles were recorded by counting from each plant in the pot. Grains of each panicle from each plant in the pot were also counted and averaged**.** Panicle length was calculated from the neck node to the tip of the uppermost spikelet. Average length was calculated in cms. Yield was recorded following threshing and drying. Plant Nutrient uptake was recorded by collecting plant samples after harvest. Afterwards these were placed in paper bags made of butter paper and dried in hot air oven at 60 °C. Further they were grinded to fine powder and placed in paper bags for determining N, P, K contents. Grinded plant samples (0.5–1 g) were digested by mixing with concentrated HNO_3_ and HClO_4_ (4:1) for estimating P and K contents. Concentrated H_2_SO_4_ and digestion mixture as suggested by Jackson (1973) were used to estimate Nitrogen (N).

### 2.4 Determination relative efficiency of phosphorous use (REP%)

REP% was determined as the ratio between the plant dry mass (DM) under low Pi (Phosphate) and under high Pi, (Kshetri et al., 2018, Ozturk et al., 2003).

### 2.5 Statistical analysis

The data recorded was subjected to statistical analysis at 5% level of significance (P = 0.05) using OPSTAT software (Gomez and Gomez, 1984, Sheoran et al., 1998).

## 3 Results

### 3.1 Effect of PSRB isolates along-with TCP on available nutrients (N,P,K) in soil at growth stages of rice crop

**Fig. 1:**
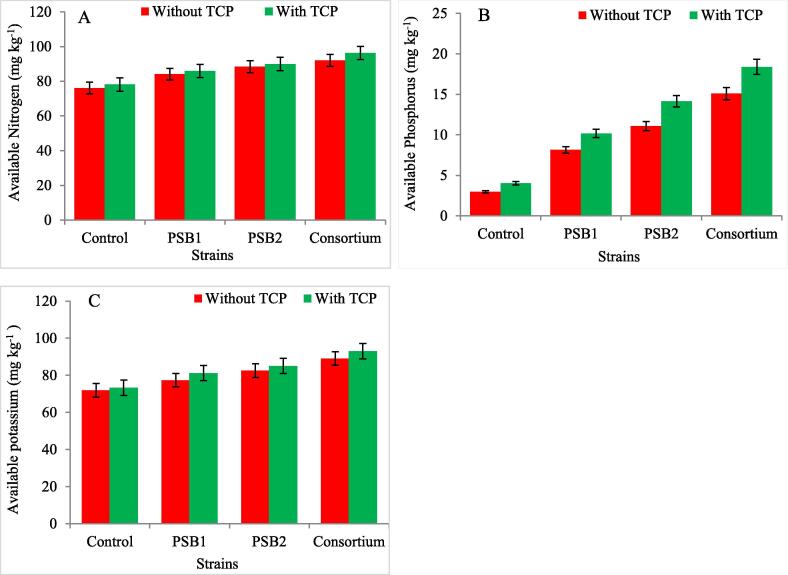
Effect of PSRB inoculation along-with TCP on available nutrients A: Nitogen (N); B: Phosphorus (P) and C: Potassium (K) in soil at growth stages of rice crop.

Soil Nitrogen, Phosphorous and Potassium (K), are an indicators of the amount of availability of these nutrient for plant uptake**.** Inoculation with PSRB isolates and TCP amended soil showed a significant difference for the availability of these nutrients at both tillering and panicle initiation stages and the availability of these nutrients were more in soil supplemented with both TCP and PSRB isolates. At both tillering and panicle initiation stages the highest available N was recorded in the treatment containing (Consortium) by 17.13% and19.1% as compared with control. In TCP amended soil highest increase for available N was recorded with TCP (1 g^−1^ soil) by 2.4% and 2.7% over control. Application of PSRB isolates also increased the P availability in soil and highest increase was recorded in the treatment containing (Consortium) by 232% and 265% compared with the control at growth stages and with TCP (1 g^−1^ soil) by 17.19% and 22.5% over control. Highest increase in available K was recorded with treatment containing (Consortium) by 19.6% and 29.2% as compared with the control and with TCP (1 g^−1^ soil) by 3.2% and 2.9% over control. There was significant interaction recorded between TCP and PSRB isolates for the available nutrients in soil at both tillering and panicle initiation stage with highest increase in the treatment containing Consortium + TCP by (21%) at tillering stage and 24% at panicle initiation stage for available N, available P by (355%) at tillering stage and (413%) at panicle initiation stage and 27.5% at tillering stage and 31.7% at panicle initiation stage for the available K in soil. Decrease in the available nutrients in soil was recorded at harvest stage with both TCP and PSRB isolates as depicted in (Table 1 and Fig. 1).

**Table 1:** Effect of PSRB inoculation along-with TCP on N, P, K availability at growth stages of rice crop.

	Available Nitrogen (mg kg^−1^)			Available Phosphorus (mg kg^−1^)			Available Potassium (mg kg^−1^)		
Treatments	Tillering stage	P.I stage	Harvest Stage	Tillering Stage	P.I stage	Harvest Stage	Tillering Stage	P.I stage	Harvest stage
TCP									
SEm(±)	0.21	0.20	0.23	0.21	0.20	0.17	0.21	0.20	0.21
CD	0.63	0.62	0.68	0.61	0.60	0.52	0.62	0.60	0.63
Isolates									
SEm(±)	0.30	0.29	0.32	0.29	0.28	0.24	0.29	0.28	0.30
CD	0.89	0.88	0.96	0.87	0.85	0.73	0.88	0.85	0.89
F probability Test									
P(TCP)	0.63*	0.62*	0.68*	0.61*	0.60*	0.52*	0.62*	0.60*	0.63*
B(PSRB)	0.89*	0.88*	0.96*	0.87*	0.85*	0.73*	0.88*	0.85*	0.89*
P*B	1.26*	1.23*	1.36*	1.23*	1.21*	1.04*	1.24*	1.21*	1.26*

* Significant at P < 0.05.

### 3.2 Effect of PSRB inoculationalong-with TCP on biological activity in soil at growth stages of rice crop

**Fig. 2:**
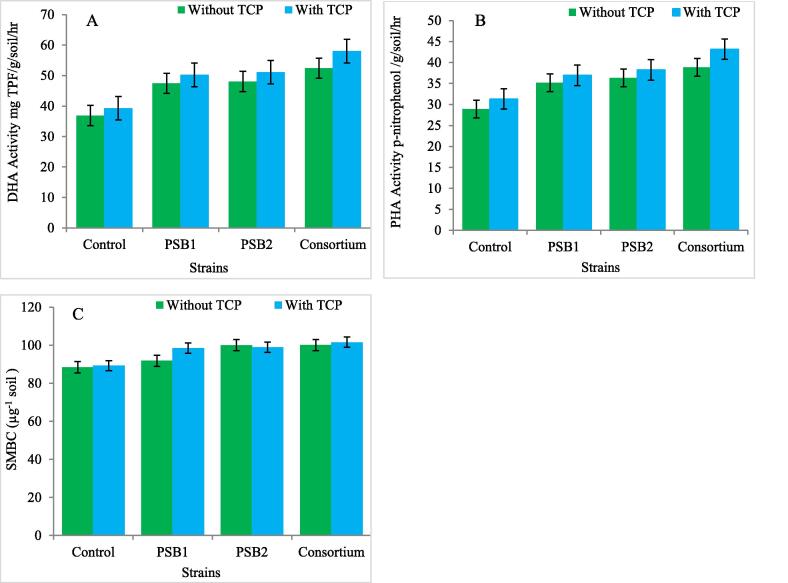
Effect of PSRB inoculation along-with TCP on biological activity in soil at growth stages of rice crop. A: DHA Activity mg TPF/g/soil/hr B: PHA Activity p-nitrophenol /g/soil/hr C: SMBC (µg-1 soil).

Biological activity of the soil is indicated by Microbial biomass present. It also describes the portion of the soil which is accountable for cycling of nutrients and energy with respect to the regulation of transformation of the organic matter. It was utilized in the current study inorder to find out effect of inoculated bacteria. Enzymes such as Phosphatase and Dehydrogenase found in soil were also utilized in the present study as natural markers to determine the activities of inoculated bacteria in the potted soil. Application of PSRB isolates increased the microbial biomass carbon in soil and highest increase was recorded in the treatment containing (Consortium) by 16.4% and 16.5% as compared with control at both tillering and panicle initiation stages and with TCP (1 g^−1^ soil) by 2.2% and 2.4% over control. Highest increase in dehydrogenase activity was recorded with treatment containing (Consortium) by 34.7% and 43.8% as compared with control and with TCP (1 g^−1^ soil) by 6.7% and 6.6% over control at growth stages of crop. Significant interaction between TCP and PSRB isolates for microbial biomass carbon in soil and dehydrogenase activity in soil with highest increase recorded in treatment comprising of Consortium + TCP with 26.05% at tillering stage and 24.6% at panicle initiation stage for microbial biomass carbon in soil and with 44.6% at tillering stage and 55.07% at panicle initiation stage for dehydrogenase activity in soil. Decrease in both microbial biomass carbon and dehydrogenase activity in soil was recorded at harvest stage. Phosphatase activity in soil was decreased at growth stages with the addition of PSRB isolates and TCP in soil as depicted in (Table 2 and Fig. 2).

**Table 2:** Effect of PSRB inoculation along with TCP on biological activity in soil at growth stages of rice crop.

	SMBC ((µg^−1^soil)			DHA Activity µg-TPFg^−1^soil hr^−1^)			PHA Activity (µg- PNP g^−1^soil hr ^−1^)		
Treatments	Tillering stage	P.I stage	Harvest stage	Tillering stage	P.I stage	Harvest stage	Tillering stage	P.I stage	Harvest stage
TCP									
SEm(±)	0.66	0.69	0.60	0.20	0.18	0.18	0.21	0.21	0.21
CD	1.99	2.07	1.79	0.60	0.55	0.55	0.62	0.62	0.64
Isolates									
SEm(±)	0.94	0.98	0.08	0.28	0.26	0.26	0.29	0.29	0.30
CD	2.82	2.93	2.53	0.85	0.78	0.78	0.87	0.88	0.90
F probability Test									
P(TCP)	1.99*	2.07*	1.79*	0.60*	0.55*	0.55*	0.62*	0.62*	0.64*
B(PSRB)	2.82*	2.93*	2.53*	0.85*	0.78*	0.78*	0.87*	0.88*	0.90*
P*B	3.99*	4.15*	3.58*	1.21*	1.11*	1.11*	1.24*	1.25*	1.27*

* Significant at P < 0.05.

### 3.3 Effect of PSRB isolates along-with TCP on yield attributes and nutrient uptake in rice crop

**Fig. 3:**
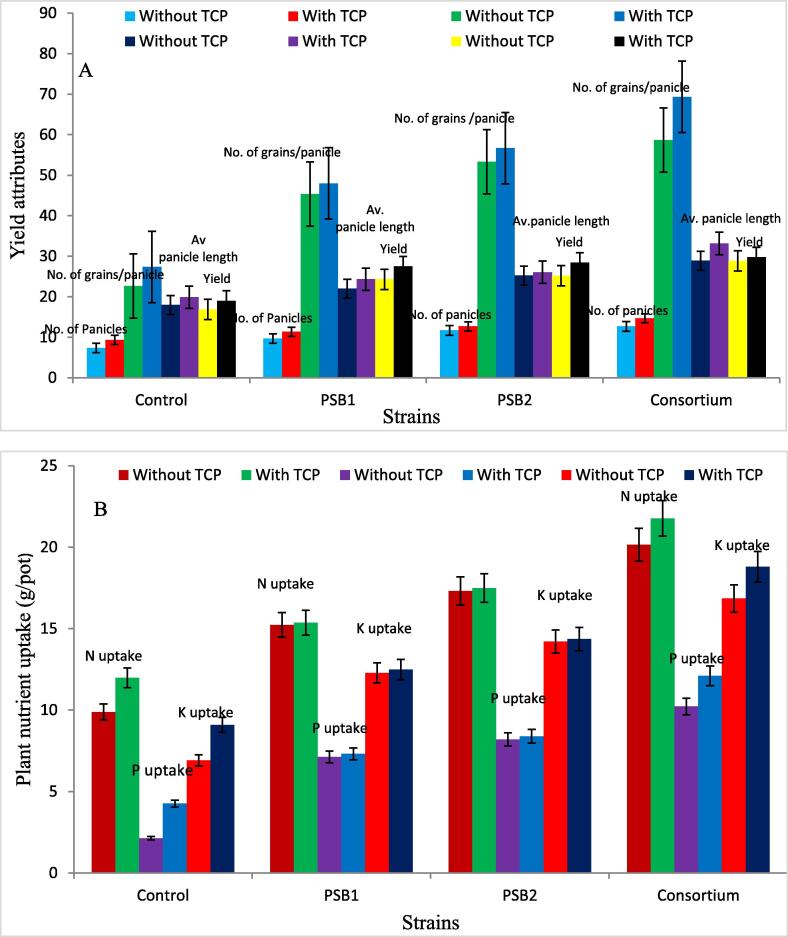
Effect of PSRB inoculation along-with TCP on A: yield attributes and B: nutrient uptake of rice crop.

Application of PSRB isolates increased the yield and other yield related parameters of rice crop and highest increase in number of panicles^−1^ plant (54.8%), number of grains^−1^ panicle (156%), average panicle length (63.9%) and yield (63.2%) (g/pot) was observed in treatment containing (Consortium) as compared with control and with TCP(1 g^−1^ soil) increase in no. of panicles^−1^ plant (16.1%), no. of grains^−1^ panicle (11.8%) average panicle length( 9.8%) and yield (g/pot) (9.9%) was observed as compared with control. Significant interaction was recorded between TCP and PSRB isolates for the yield and yield attributes in rice crop. Highest increase was observed in treatment comprising of Consortium + TCP,54.7% increase in panicles^−1^ per plant, 205% in no. of grains^−1^ panicle, 84.3% in average panicle length and 76.9% in yield. Highest increase in total N, P, K uptake of rice crop was observed in treatment containing consortium to 91.7%, 248%, and 122% respectively as compared with control. Significant interaction was recorded between TCP and PSRB isolates for the nutrient uptake in rice crop with highest increase in nutrient uptake recorded for treatment containing Consortium + TCP with 120% increase in N uptake, 165% in P uptake and 172% in K uptake as depicted in Table 3 and Fig. 3.

**Table 3:** Effect of PSRB inoculation and TCP on yield attributes and uptake of nutrient in rice crop.

Treatments	No. of panicles^−1^plant	No. of grains^−1^panicle	Average panicle length(cm)	Yield(g^−1^ pot)	N uptake (g/plot)	P uptake (g/plot)	K uptake (g/plot)
TCP N uptake (g/plot)							
SEm(±)	0.24	0.50	0.20	0.20	0.18	0.19	0.21
CD	0.73	1.51	0.60	0.61	0.56	0.58	0.62
Isolates							
SEm(±)	0.34	0.71	0.29	0.28	0.26	0.28	0.29
CD	1.03	2.13	0.86	0.87	0.79	0.83	0.87
F probability Test							
P(TCP)	0.73*	1.51*	0.60*	0.61*	0.56*	0.58*	0.62*
B(PSRB)	1.03*	2.13*	0.86*	0.87*	0.79*	0.83*	0.87*
P*B	NS	3.02*	1.21*	1.23*	1.11*	1.17*	1.23*

* Significant at P < 0.05.

### 3.4 Effect of PSRB isolates and TCP on relative efficiency of phosphorus use (%).

Addition of PSRB isolates increased REP% and highest REP% was observed in the treatment comprising of both the isolates by 86.3% as compared with control whereas with TCP (1 g^−1^ soil) REP% was increased by 79.3% over control as depicted in (Table 4).

**Table 4:** Effect of PSRB inoculation and TCP on relative efficiency of phosphorus use (%) of rice crop.

	Dry matter (g/plant)		
Treatments	Low Pi	High Pi	REP%
P_0_ (0 g^−1^ soil)	0.48	0.89	53.9%
P_1_ (1 g^−1^ soil)	1.42	1.79	79.3%
B_0_ (Control)	1.28	1.65	77.5%
B_1_ (PSRB 1)	2.73	3.30	82.7%
B_2_ (PSRB 2)	3.83	4.47	85.7%
B_3_ (PSRB 3)	3.97	4.60	86.3%

### 3.5 Relationship between available soil nutrients, enzyme activity and plant nutrient uptake

Data from (Table 5) depicted that soil available phosphorus is positively correlated with soil available nitrogen(N) (1.00). Soil available potassium had a positively correlation with soil available N (0.99) and soil available phosphorus (0.99). Plant nitrogen uptake is positively correlated with soil available nitrogen (0.99), soil available phosphorus (0.98) and soil available potassium (0.98). Plant phosphorus uptake was also found to be correlated with available soil nitrogen (0.99), phosphorus (0.98) and potassium (0.97) and plant nitrogen uptake positively (0.99). It was also found that plant potassium uptake is positively correlated with soil available nitrogen (0.99), soil available phosphorus (0.98), soil available potassium (0.98), plant nitrogen uptake (1.00) and plant phosphorus uptake (1.00). Soil microbial biomass carbon was also found positively correlated with soil available nitrogen (0.98), soil available phosphorus (0.98), soil available potassium (0.96), plant nitrogen uptake (0.97), plant phosphorus uptake (0.98) and plant potassium uptake (0.97). Dehydrogenase activity showed a positive correlation with available soil nitrogen (0.97), phosphorus (0.98) and potassium (0.95), plant nitrogen uptake (0.96), plant phosphorus uptake (0.98), plant potassium uptake (0.96) and soil microbial biomass carbon (0.99). Phosphatase activity was found positively correlated with soil available nitrogen (0.98), soil available phosphorus (0.98), soil available potassium (0.97), plant nitrogen uptake (0.97), plant phosphorus uptake (0.98), plant potassium uptake (0.98), soil microbial biomass carbon (0.97) and dehydrogenase activity (0.99). Yield showed a positive correlation with available soil nitrogen (0.95), phosphorus (0.95), and potassium (0.92). Plant nitrogen uptake (0.93), plant phosphorus uptake (0.94), plant potassium uptake (0.93), soil microbial biomass carbon (0.97), dehydrogenase activity (0.98) and phosphatase activity (0.96) also were positively correlated to the yield.

**Table 5:** Coefficient of correlation between available soil nutrients, enzyme activity and plant nutrient uptake.

	SoilAv.N	SoilAv.P	SoilAv.K	Plant N uptake	Plant P uptake	Plant K uptake	SMBC	DHA	PHA	Yield
Soil Av.N	1									
SoilAv.P	1.00	1								
SoilAv.K	0.99	0.99	1							
Plant N uptake	0.99	0.98	0.98	1						
Plant P uptake	0.99	0.98	0.97	0.99	1					
Plant K uptake	0.99	0.98	0.98	1.00	1.00	1				
SMBC	0.98	0.98	0.96	0.97	0.98	0.97	1			
DHA	0.97	0.98	0.95	0.96	0.98	0.96	0.99	1		
PHA	0.98	0.98	0.97	0.97	0.98	0.98	0.97	0.99	1	
Yield	0.95	0.95	0.92	0.93	0.94	0.93	0.97	0.98	0.96	1

DHA = Dehydrogenase activity, SMBC = Soil microbial biomass carbon, PHA = Phosphatase activity.

## 4 Discussion

### 4.1 Effect of PSRB isolates along-with TCP on available N, P and K in soil at growth stages of rice crop

Inoculation with PSRB isolates and TCP amended soil showed a significant difference for the availability of these nutrients at both tillering and panicle initiation stages and the availability of these nutrients were more in soil supplemented with both TCP and PSRB isolates. At both tillering and panicle initiation stages the highest available N was recorded in the treatment containing (Consortium) by 17.13% and 19.1% as compared with control. In TCP amended soil highest increase for available N was recorded with TCP (1 g^−1^ soil) by 2.4% and 2.7% over control. These findings were equally supported by the observations of Gulati et al., 2010 who also found the similar pattern of nutrient availability during different consortiums for cold deserts of trans Himalayas. Application of PSRB isolates also increased the availability of P in soil (Sharma et al., 2013, Zahid et al., 2015, Zhang et al., 2017). Highest increase was reported in the treatment containing (Consortium) by 232% and 265% compared with the control at growth stages and with TCP (1 g^−1^ soil) by 17.19% and 22.5% over control. The outcomes are in line with the findings of Sundara et al., (2002). Highest increase in available K was recorded with treatment containing (Consortium) by 19.6%, and 29.2% as compared with the control and with TCP (1 g^−1^ soil) by 3.2% and 2.9% over control. Such results were also reported by Vyas and Gulati (2009) in maize. There was significant relation recorded amid TCP and PSRB isolates for the available nutrients in soil at both tillering and panicle initiation stages (Chakkravarthy et al., 2010). There was highest increase in the treatment containing Consortium + TCP (21%) at tillering stage and 24% at panicle initiation stage for available N, available P (355%) at tillering stage and (413%) at panicle initiation and 27.5% at tillering stage and 31.7% at panicle initiation stage for the available K in soil. Decrease in the available nutrients in soil was recorded at harvest stage with both TCP and PSRB isolates as depicted in Table 1.

### 4.2 Effect of PSRB isolates along-with TCP on biological activity at growth stages of rice crop in soil

The biological properties of the soil are indicated by availability of microbial biomass carbon (Bach et al., 2020, Liddle et al., 2020). It also indicates the proportion of the soil liable for the energy and nutrient cycling with the direction of organic matter transformation which was utilized in our study to find out the effect of inoculated bacteria. Dehydrogenase and Phosphatase enzymes found in soil were also utilized in the present study as biological markers to find out the behavior of inoculated bacteria in the pot soil. Application of PSRB isolates increased the microbial biomass carbon in soil and highest increase was recorded in the treatment containing (Consortium) by 16.4% and 16.5% as compared with control at both tillering and panicle initiation stages and with TCP (1 g^−1^ soil) by 2.2% and 2.4% over control as also reported by Raghuveer et al., 2017. Highest increase in dehydrogenase activity was recorded with treatment containing (Consortium) by 34.7% and 43.8% as compared with control and with TCP (1 g^−1^ soil) by 6.7% and 6.6% over control at growth stages of crop. Similar kind of findings was reported by Stephen et al., 2015, Raghuveer et al., 2017 in their respective experminents. Significant interaction between TCP and PSRB isolates for microbial biomass carbon in soil and dehydrogenase activity in soil with highest increase 26.05% recorded in treatment comprising of Consortium + TCP at tillering stage and 24.6% at panicle initiation stage for microbial biomass carbon in soil and with 44.6% at tillering stage and 55.07% at panicle initiation stage for dehydrogenase activity in soil respectively. Decrease in both microbial biomass carbon and dehydrogenase activity within the soil was recorded at harvest stage. Phosphatase activity in soil was decreased at growth stages with the addition of PSRB isolates and TCP in soil. Similar findings were also reported by Sreelakshmi and Aparna (2019) as depicted in Table 2.

### 4.3 Effect of PSRB isolates along-with TCP on yield attributes and nutrient uptake in rice crop

Yield and yield related parameters of rice were increased following the application of PSRB and highest increase in number of panicles^−1^ plant (54.8%), number of grains^−1^ panicle (156%), average panicle length (63.9%) and yield (63.2%) (g/pot) was observed in treatment containing (Consortium) as compared with control and with TCP(1 g^−1^ soil) increase in no. of panicles^−1^ plant (16.1%), no. of grains^−1^ panicle (11.8%) average panicle length (9.8%) and yield (g/pot) (9.9%)  was observed as compared with control. Significant interaction was recorded between TCP & PSRB isolates for the yield and yield attributes in rice crop. Highest increase was observed in treatment comprising of Consortium + TCP with 54.7% increase in no. of panicles^−1^ plant, 205% in no. of grains^−1^ panicle, 84.3% in average panicle length and 76.9% in yield. Similar kinds of results were reported by various scientists (Lavakush et al., 2014, Deshwal and Kumar, 2013, Hameeda et al., 2006). Highest increase in total N, P, K uptake of rice crop was recorded in treatment containing Consortium with 91.7%, 248% and 122% as compared with control. Significant interaction was recorded between TCP and PSRB isolates for the nutrient uptake in rice crop with highest increase in nutrient uptake recorded for Consortium + TCP with 120% increase in N uptake, 165% in P uptake and 172% in K uptake. The same were also reported by Viruel et al., 2014 as depicted in Table 3.

### 4.4 Effect of PSRB isolates and TCP on relative efficiency of phosphorus use (%)

Addition of PSRB isolates increased REP% by 86.3%  and highest REP% was observed in the treatment comprising of both the isolates as compared with control and with TCP (1 g^−1^ soil) REP% was increased by 79.3% over control as also reported by Kshetri et al., 2018 as depicted in Table 4.

### 4.5 Relationship between available soil nutrients, enzyme activity and plant nutrient uptake

In the present investigation the correlation coefficients were worked out for all the parameters and all the combinations were significantly and positively correlated with each other (Table 5). In the present experiment soil parameters like soil available N, P and K showed a positive correlation with plant nitrogen, phosphorus and potassium uptake, soil microbial biomass carbon, dehydrogenase activity, phosphatase activity and yield. These results are in accordance with Elhaissoufi et al. (2020) who observed a positive and significant correlation between the soil and plant parameters, which might be due to incorporation of PSRB inoculants that increases the availability of nutrient to crop, through improvement in root nutrient acquisition and absorptive capacity thus enhancing the soil as well as plant nutrient status. It was also reported by many workers that phosphatase enzyme activity is in direct correlation with activity of PSRB in soil that improved nutrients obtained from soil (i.e. C, N, and P) through increased activities of alkaline phosphatase, invertase and dehydrogenase, which lead to plant growth promotion (Majumder and Das, 2016, Xu et al., 2018).

## 5 Conclusions

The observation recorded during the present investigation revealed the superiority of the inoculated PSRB isolates isolated from the rice rhizosphere as P solubilizers and in making the availability of nutrients in the soil easy when applied with TCP. It leads to enhancement in the traits promoting plant growth. Enhanced growth within the plants due to TCP solubilization revealed that phosphate solubilization is an important mechanism of plant growth promotion by the bacterial strain (PSRB). The availability of nutrients in plant growth promotion revealed that TCP is easily soluble by the PSRB isolates which increased the available phosphorus content in soil. Bacteria proliferate more with this phosphorus source and also due to extensive root system of rice and its improvement made the roots absorb the nutrients at larger depth and increased the uptake which in turn proved beneficial for reproductive organs of rice crop. These results implied potential application of the PSRB strains as an important bioinoculants in phosphorus-deficient soils by showing high P-fixing capacity. However, more extensive research needs to be done for the identification of PSRB isolates which are still unidentified and are crop as well as site-specific.

## 6 Ethics approval

Not applicable.

## 7 Consent to participate

All authors consent to participate in this manuscript.

## 8 Consent for publication

All authors consent to publish this manuscript in Saudi Journal of Biological Science.

## 9 Availability of data and material

Data will be available on request to corresponding or first author.

## 10 Code availability

Not applicable.

## Author contributions

RG and AK drafted the experimental design and performed the experiments. RG and AK helped in data collection, data analysis and initial draft of manuscript text. All authors read the manuscript before communication.

## Declaration of Competing Interest

The authors declare that they have no known competing financial interests or personal relationships that could have appeared to influence the work reported in this paper.
